# Association between no-shows to scheduled clinic appointments and 30-day risk of overdose in patients prescribed methadone for opioid use disorder

**DOI:** 10.1371/journal.pone.0329067

**Published:** 2025-10-27

**Authors:** Henry Kaufman Philofsky, Ian Cero, Daniel D. Maeng, Myra L. Mathis

**Affiliations:** Department of Psychiatry, Strong Memorial Hospital, Rochester, New York, United States of America; Virginia Mason Franciscan Health, UNITED STATES OF AMERICA

## Abstract

**Background:**

The introduction of synthetic opiates and non-opiate sedatives into the illicit drug market has increased overdose risk for individuals who use opiates and other drugs. The ongoing risk of overdose for patients receiving methadone as a medication for opioid use disorder in the context of this more potent and less predictable drug supply is not well characterized. Additionally, little research has explored whether commonly available clinical data (including data available even in low resource settings) can predict near-term acute overdose in patients prescribed methadone for opioid use disorder.

**Objective:**

To determine whether the number of recent no-shows to scheduled clinic appointments in the past 30 days is associated with 30-day overdose risk among patients enrolled in one Opioid Treatment Program who are prescribed methadone for opioid use disorder.

**Methods:**

We analyzed clinical records from 1,049 patients in an opioid treatment program (May 2020–April 2024), and for each patient-day, counted the number of no-shows to scheduled clinic appointments (not methadone administrations) in the previous 30 days. Associations between the number of standardized no-shows in the past 30 days and overdose in the subsequent 30 days were analyzed with logistic regression via generalized linear model controlling for temporal and patient-specific variables. Goodness of fit was assessed with marginal R^2^ and a simulation-based approach designed for multilevel models.

**Results:**

The sample included 56 overdoses with an average of 0.919 no-shows in the last 30 days (std. dev 1.37). The z-standardized number of no-shows to scheduled appointments in the past 30 days was both statistically and clinically significantly associated with risk of overdose in the next 30 days adjusting for study month and season (odds ratio 1.18 [95% CI 1.13–1.23] P < 0.001), as well as adjusting for demographics and overdoses during study period (odds ratio 1.28 [95% CI 1.22–1.34] P < 0.001), and a marginal R^2^ of 0.04. Model diagnostics revealed adequate fit using a generalized additive model, with results virtually unchanged from the generalized linear model.

**Conclusion:**

No-shows to scheduled clinic appointments in the past 30 days are significantly associated with overdose risk in the next 30 days with a linear relationship among patients receiving methadone in a single opioid treatment program. Population-specific acute risk prediction tools could help clinicians prioritize resources for timely intervention.

## Introduction

The rates of opioid overdose have increased since the introduction of synthetic opiates and non-opiate sedatives into the American illicit drug market [1]. Patients receiving methadone for opioid use disorder have high rates of concomitant fentanyl misuse [2,3]. There has been substantial research into prediction of opiate-related overdose [4–6], with some research on the risk of overdose for patients prescribed methadone for opioid use disorder [7–10]. However, most opioid overdose prediction research has focused on long time horizons (e.g., 6–12-month risk episodes), which is not helpful in guiding acute interventions.

In response, this study is part of an ongoing effort to identify factors acutely associated with overdose risk within a single opioid treatment program to guide intervention resources (e.g., mobile crisis team visits, peer specialist outreach). Previous research has shown that no-shows to scheduled appointments can be a meaningful signal of risk across a range of conditions [10]. Since COVID-19, we expect this finding to extend to patients taking methadone for opioid use disorder as part of Opioid Treatment Programs. Prior to COVID-19, patients in OTPs were required to meet with substance abuse counselors to continue to receive Methadone. After the start of the pandemic, on May 19, 2020, regulators changed this requirement to be optional. As such, after May 19, 2020, patients in OTPs who scheduled visits with clinicians have an actual clinical need to be addressed. Therefore, missing these appointments may not only be a signal of other factors that could increase risk of overdose that could be associated with no-showing to an appointment (such as substance use), but that patients that no-show to appointments also miss the benefits of clinical appointments directly. While chronic risk factors for overdose have been explored (see above), and clinic attendance has been tied to negative outcomes on a chronic basis, the acute relationship between clinic attendance and overdose risk has not yet been explored, including in patients taking methadone for opioid use disorder. We aim to close this gap by testing the hypothesis that recent no-shows to scheduled clinic visits are acutely associated with increased overdose risk. To our knowledge, this is the first study examining the acute risk of overdose among patients prescribed methadone for opioid use disorder, which could be an important progression in this research as acute risk factors for overdose have not been well explored in this patient population, and knowledge of acute risk factors such as no-shows to scheduled clinic appointments in the past 30 days could enable a more principled allocation of resources to intervene on an acute basis.

## Methods

### Study population

The study included patients treated with methadone at an Opioid Treatment Program between May 19, 2020, and April 30, 2024. The start date corresponds to the relaxation of attendance rules in the program due to regulatory action, while the end date marks the study’s cutoff. This sample therefore reflects the maximum possible sample we could realistically achieve within the current regulatory framework with above minimal risk of overdose [11]. The sample includes all individuals enrolled in the OTP who had received at least one methadone dose within the preceding 30 days, as patients who go more than 30 days without a dose are discharged from the program. Patients were only included in the sample for time periods when they received at least one dose in the preceding 30 days.

### Ethical considerations

The Research Subject Review Board (RSRB) at the University of Rochester Medical Center determined that this study met federal and University criteria for exemption as secondary research on data or specimens (study ID STUDY00009188). Data was accessed on 10/31/2024 and was de-identified prior to analysis. Only one author (H.K.P.) maintained access to the identification key for data validation purposes.

### Measures

#### Overdose and no-shows.

The primary outcome was an overdose within 30 days, defined as a binary variable indicating, for each patient for any day (index day) in the data set, whether the index day preceded an overdose event of any type by 30 days or less. The risk period of 30 days is widely used outside of addiction research [12–15], has previously been used in overdose research [16], and was chosen because it is a timescale that is compatible with acute clinical interventions. Then, for each index day, we counted the number of no-shows to scheduled appointments with clinicians over the previous 30 days. A “no-show” was defined as failure to appear for any scheduled appointment with clinical staff (counselors, medical providers, case managers etc.) without prior cancellation or rescheduling; this does not include no-shows to methadone administration appointments. Each count of no-shows for every patient for each day included in the sample was then standardized compared to all patients’ 30-day no-show counts before the index day using a Z-score. As such, on any given day in the sample, each patient’s count of no shows in the past 30 days includes only their own data; then that count is Z-scored using all patient’s counts for the days before that particular day, which avoids future contamination. Clinic appointment attendance data and methadone prescribing data were obtained from the program’s electronic health record (EHR), while overdose events were identified through the program’s critical event reporting system.

### Patient characteristics

Patient-level characteristics used as covariates in the multivariate model included age, sex, race/ethnicity, and number of prior overdoses as standard predictors based on overdose risk literature [10]. Race/ethnicity was dichotomized (White or Caucasian vs. non-White) due to the infrequent representation of certain racial and ethnic categories, which could affect model stability.

### Temporal characteristics

Study month and season were included to account for potential temporal confounders that could influence appointment attendance such as seasonal change, program level changes, and changes in clinician population, as well as potential changes in the street market of narcotics that could influence risk of overdose [17].

### Missing data

Missing data was not an issue for attendance data, nor the other covariates, as these are structured data that are required of each patient receiving treatment through our Opioid Treatment Program. However, it’s likely that not all overdoses that occurred within the population were reported as the critical event reporting system only includes events reported by patients and logged by clinicians into the reporting system, which introduces multiple potential points for data loss.

### Statistical modeling

#### Statistical model.

Mixed-effects logistic regression was used to model the association between the number of no-shows to scheduled appointments in the past 30 days (predictor) and the risk of overdose in the subsequent 30 days (outcome). Multi-level modeling with individual patient-level intercepts (random effects) was used to control for individual variability in overdose risk using a generalized linear mixed model. We conducted two analyses on the z-standardized number of no-shows to assess the directionality and magnitude of the relationship: controlling for temporal variables only and controlling for both temporal and patient characteristics. The latter analysis was repeated using a generalized additive model to incorporate non-linear effects of study month and age. To convert the standardized regression coefficient for missed appointments to an estimate of the marginal effect of each additional no-show, we applied an inverse standardization transformation to the odds ratio. This transformation assumes the average of historical standardization parameters across the study period as the historical means and standard deviations used in the z-standardization calculation evolved over time. The evolution of these parameters was assessed with coefficient of variation. We applied the same transformation to the upper and lower bounds of the confidence interval to obtain confidence intervals.

### Diagnostics and sensitivity analyses

All regressions used two-tailed tests at an alpha level of 0.05. Multicollinearity was assessed using generalized variance inflation factors adjusted for degrees of freedom, and the linearity of the relationship between the variable of interest and the outcome was assessed using a general additive model with splines. Goodness of fit was assessed in two ways. First, the marginal R^2^ was calculated to determine the variance explained by fixed effects. Second, a simulation-based approach specific to multi-level models was used to analyze the correctness of the residual distribution and the degree to which the variance of the data is properly captured in the statistical model using visual inspection as well as the Kolmogorov-Smirnov test and dispersion tests respectively [18].

### Software and tools

Data preprocessing and analysis were conducted in R version 4.4.2 using the glmTMB mgcv, dplyr, performance, and tidyr, DHARMa packages.

## Results

### Descriptive analysis

The dataset comprised 828,154 daily observations from 1,049 patients, during which 56 overdoses occurred. The global level mean number of no-shows to appointments in the past 30 days was 0.919 (SD = 1.37) (see Table 1 for descriptive statistics).

**Table 1:** Descriptive statistics.

Category	Proportion/Mean (SD)
Global mean no-shows in past 30 days	0.919 (1.37)
Female	40.54%
Age	45.55 (12.40)
Number of overdoses	56
Number of patients who overdosed	49
Percentage of daily observations within 30 days of overdose in sample	0.20%
White or Caucasian	71.42%
Black or African American	9.27%
Other	14.34%
Multiracial	3.60%
Unknown	0.39%
Patient Refused	0.07%
American Indian or Alaskan Native	0.68%
Asian	0.28%
Native Hawaiian or Other Pacific Islander	0.05%

### Mixed effects logistic regression analysis

The z-standardized number of no-shows to scheduled appointments in the past 30 days was both statistically and clinically significantly associated with risk of overdose in the next 30 days adjusting for temporal variables (odds ratio 1.18 [95% CI 1.13–1.23] P < 0.001) (Table 2). The magnitude of the effect increased after the addition of patient characteristics (odds ratio 1.28 [95% CI 1.22–1.34] P < 0.001). Only study month, season, and cumulative overdoses during study period were significant among control variables (Table 3). The historical standardization parameters evolved over the study period, with historical means averaging 0.837 missed appointments in the past 30 days with standard deviation of 1.329, with coefficients of variation of 18.4% and 6.5% respectively. Using the historical standard deviation to invert the standardization of the regression coefficient for missed appointments in the past 30 days, each additional missed appointment in the past 30 days was associated with 20% higher odds of overdose in the next 30 days (odds ratio 1.20 [95%CI 1.16–1.24]).

**Table 2:** Logistic regression results for multivariate model with z-standardized no-shows in the past 30 days adjusting for season and study month.

Variable	Odds Ratio	Lower 95% CI	Upper 95% CI	P-Value
(Intercept)	0	0	0	< 0.001
Z-scored no-shows in the past 30 days	1.18	1.13	1.23	< 0.001
Season: Spring	0.42	0.35	0.5	< 0.001
Season: Summer	1.52	1.33	1.72	< 0.001
Season: Winter	0.73	0.64	0.85	< 0.001
Study month	1.05	1.05	1.06	< 0.001

**Table 3:** Logistic regression results for multivariate model with z-standardized no-shows in the past 30 days adjusting for season, study month, z-standardized age, patient sex, and patient race/ethnicity (dichotomized).

Variable	Odds Ratio	Lower 95% CI	Upper 95% CI	P-Value
(Intercept)	0	0	0	< 0.001
Z-scored no-shows in the past 30 days	1.28	1.22	1.34	< 0.001
Season: Spring	0.3	0.25	0.37	< 0.001
Season: Summer	1.2	1.03	1.39	< 0.001
Season: Winter	0.74	0.63	0.86	< 0.001
Study month	1.28	1.26	1.29	< 0.001
z-score Age	1.05	0.34	3.23	0.926
Patient Sex = Male	1.19	0.13	11.05	0.882
Race/Ethnicity = white	1.19	0.1	14.08	0.889
Patient-specific prior overdoses during study period	0.01	0.01	0.01	< 0.001

### Diagnostics and sensitivity analyses

**Fig 1:**
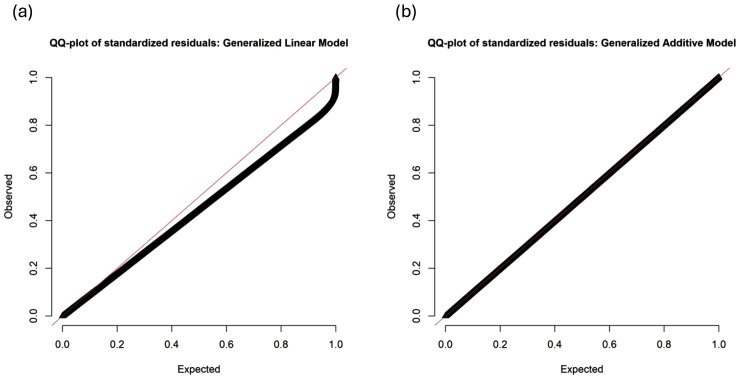
DHARMa diagnostic plots for model comparison. Quantile-quantile plots of standardized residuals from (A) generalized linear mixed model and (B) generalized additive model approaches. The generalized linear model (Panel A) displays systematic deviations from the expected uniform distribution diagonal line) indicating model misspecification while the generalized additive model (Panel B) shows superior fit with residuals closely following the expected uniform distribution.

The marginal R^2^ of the model including both temporal variables and patient characteristics was 0.04. Generalized variance inflation factors adjusted for degrees of freedom for both multivariate analyses were below 1.01, indicating minimal multicollinearity. Spline analysis using a generalized additive model revealed statistically significant effects supporting a generally linear relationship between the number of no-shows and 30-day risk of overdose, with a non-linear slope at the extreme of standardized no-shows. However, simulation-based diagnostics of the generalized linear model that included both patient characteristics and temporal characteristics revealed a significant Kolmogorov-Smirnov test with significant indicating poor residual uniformity and excessive under dispersion. The analysis was repeated using a generalized additive model with study month and age modeled with splines, and simulation-based diagnostics revealed adequate model specification (Kolmogorov-Smirnov test p = 0.1277), with mild under dispersion (ratio 0.899, p < 0.001), consistent with conservative variance estimates. The quantile-quantile plots of the standardized residuals from both the generalized linear and generalized additive models are shown in Fig 1. The estimate of the effect of no-shows on overdose risk was virtually unchanged in the generalized additive model results compared to the results of the generalized linear model.

## Discussion

Recent no-shows to scheduled appointments in the past 30 days appear to be strongly associated with overdose risk in the subsequent 30 days in a generally linear fashion. These findings demonstrate that tracking dynamic features may help prioritize high-risk patients for targeted interventions. Importantly, these data are also readily accessible even to low resource clinics due to existing regulatory requirements. This study is the first, to our knowledge, to examine how recent appointment attendance predicts acute risk of overdose among patients prescribed methadone for opioid use disorder.

These results show that each additional missed appointment is associated with approximately 20% higher odds of overdose in the next 30 days. While no-shows may not directly increase the risk of overdose, no-shows may be caused by the same factors that increase the risk of overdose, such as substance use or psychosocial stress. This study has clear and actionable implications for reducing overdose in our clinic: tracking clinic appointment attendance patterns and identifying high-risk patients based on no-shows could enable targeted outreach and reduce risk of patient overdoses.

### Limitations

The study’s observational nature precludes causal inferences, as the associations identified may reflect underlying unmeasured phenomena rather than direct effects, nor was this study designed for causal inference. These findings should therefore be interpreted as identifying potential risk factors rather than causal factors. Furthermore, this study’s reliance on the program’s critical event reporting system to identify overdoses may have underestimated the true number of overdoses, biasing results towards a null result, which suggests the true association between no-shows and acute risk of overdose may be stronger than these results suggest. Additionally, the study was conducted at a single opioid treatment program which, given the variability in how Opioid Treatment Programs are run, may limit generalizability to other programs. Especially with appointment attendance-related features, clinic-level policies related to scheduled clinic appointments (as opposed to methadone administration appointments) likely influenced the observed associations, and the exposure under consideration may not have the same meaning in other clinics. The relatively limited number of features included in the analysis, chosen for their availability and clinical relevance, also limits interpretation.

### Future directions

Future research should focus on validating these findings in new cohorts and exploring additional acute risk factors for overdose among patients prescribed methadone for opioid use disorder. This study represents an early step toward developing a risk stratification framework within opioid treatment programs. Further studies should incorporate features such as methadone dosing, urine toxicology results, laboratory data, and concurrent medications, which could enhance model precision by capturing direct measures of adherence and substance use. Additionally, prospective research should evaluate the effectiveness of implementing these features in real-time clinical decision-making, potentially improving patient outcomes in diverse treatment settings.

## Conclusion

This analysis demonstrates that recent appointment attendance patterns are significantly associated with 30-day overdose risk. These findings suggest that attendance patterns could be integrated into real-time monitoring systems to assess overdose risk and guide targeted interventions, providing a practical framework for clinic-level acute risk analysis using data collected in the normal course of clinic operations. The study highlights the feasibility of short-term overdose prediction and lays the groundwork for future research to validate these findings across diverse settings. Incorporating additional risk factors such as methadone dosing, urine toxicology, and concurrent medications may further refine prediction models, ultimately enhancing patient care and safety.
